# Emerging Trends and New Developments in Disaster Research after the 2008 Wenchuan Earthquake

**DOI:** 10.3390/ijerph16010029

**Published:** 2018-12-23

**Authors:** Cailin Wang, Jidong Wu, Xin He, Mengqi Ye, Wenhui Liu, Rumei Tang

**Affiliations:** 1 Key Laboratory of Environmental Change and Natural Disaster, Ministry of Education, Beijing Normal University, Beijing 100875, China; wangcailin@mail.bnu.edu.cn (C.W.); hxin2017@163.com (X.H.); yemengqi@mail.bnu.edu.cn (M.Y.); lwhtay1996@mail.bnu.edu.cn (W.L.); tangrumei@mail.bnu.edu.cn (R.T.); 2 State Key Laboratory of Earth Surface Processes and Resource Ecology, Faculty of Geographical Science, Beijing Normal University, Beijing 100875, China

**Keywords:** disaster, Wenchuan earthquake, bibliometric, scientific visualization, CiteSpace

## Abstract

On the tenth anniversary of the 2008 Wenchuan Earthquake, investigating the evolution of disaster science is worthwhile and can be used to improve the future execution of disaster risk management. Based on more than 55,786 articles on the relative topic of “Disaster” derived from the Web of Science Core Collection from 1999–2017, this study employs CiteSpace and Google Earth to identify and visualize the spatial distribution of publications, bursts of keywords and categories, highly cited references, and interdisciplinary levels and then identify the emerging trends of disaster research over the past 20 years. The results show that the earthquake indeed jumpstarted a massive wave of disaster research around the world and increased international cooperation over the last decade. However, in terms of both the quantity and quality of publications in disaster research fields, China is lagging behind the U.S. and European countries. Moreover, although designing disaster prevention and mitigation strategies is a new popular field of disaster science, geological environment changes and geologic hazards triggered by earthquakes are more popular research topics than disaster emergency and recovery. In addition, the transdisciplinary level of disaster science increased after the earthquake. This interdisciplinary characteristic of disaster science gradually increased in popularity, which demonstrates that people can learn from catastrophes. These emerging trends could serve as a scientific basis to clearly understand disaster science progress over the last 20 years and provide a reference for rapidly identifying frontier issues in disaster science.

## 1. Introduction

During the 21st century, disaster events, especially catastrophes, have frequently occurred and caused enormous damage [1], e.g., Hurricane Katrina in 2005 [2], the Wenchuan Earthquake in 2008 [3], and the Tohoku earthquake off the Pacific coast of Japan in 2011 [4]. These catastrophic events are often regarded as a classic case that can be used to dissect the mechanism in which disasters are triggered from specific hazards and formative environments, exposures, and vulnerabilities [5,6,7]. Additionally, the dual attributes (nature and society) of disasters ensure that their impacts are multidimensional, synthetic, and far-reaching [8]. For example, a catastrophic event could result in ecological destruction [9], environmental changes to a catchment [10], the spread of disease epidemics [11], or changes to the trajectory of regional economic growth [12]. However, it is not clear whether a catastrophic event could result in an advancement of disaster research in multiple fields or how strongly the effects of a catastrophic event are promoted. Thus, a complete review of catastrophic events is necessary to detect the contributions of such events to improving our understanding of the overall perspective of disaster research.

Furthermore, the 2008 Wenchuan Earthquake which had a magnitude of 8.0 Ms, was one of the most destructive disaster since the founding of the People’s Republic of China in 1949 [3] and evoked a massive wave of disaster research in the last decade [13,14]. However, questions remain as to how disaster research has changed (as a time node) from then until now and how wide the disparities are in disaster research worldwide. Thus, it is indispensable to review the process of disaster research in 2018, which is the tenth anniversary of the Wenchuan Earthquake.

With the rapid development of information technology over the past several decades, the scientific visualization of bibliometric analysis has been achieved. Many tools are used to investigate the evolution of scientific issues, including CiteSpace [15], VoSviewer [16], Pajek [17], and CitNetExplorer [18]. CiteSpace is the most widely applied bibliometric tool because the software is constantly updated with new functions. Moreover, the tool is also able to visualize 12 node types, i.e., Author, Institution, Country, Term, Keyword, Source, Category, Cited Reference, Cited Author, Cited Journal, Paper, and Grant. The functions of burst detection and dual-map overlays of node types are provided, which could show how a particular connection is significantly strengthened over a short period of time [15] and track the movements of scientific frontiers in terms of their footprints [19]. The latest version also provides servals interfaces for other visualizations. For example, the geospatial networks of citations could be adequately depicted by Google Earth [20]. Thus, a bibliometric analysis could be completely conducted via CiteSpace.

This article aims to monitor the trajectory and evolution of disaster research over time and identify the potential directions for future research. First, we extract 55,786 records associated with the topic “Disaster” and 3461 records about the “Wenchuan Earthquake” from the Web of Science Core Collection. Then, CiteSpace combined with Google Earth is employed to visualize four node types (Cited Reference, Keywords, Institution, and Term) of these records. Finally, the evolution of disaster research is discussed by analysing the relationships between co-cited references, the bursts of keywords and categories, the geospatial distribution of publications, and the change in citing trajectories via a dual-map overlay. All of the above results will deepen our understanding of the interdisciplinary characteristics of disaster science and can be used to better execute disaster risk management in the future. Thus, domain experts must deeply understand disaster science.

## 2. Methods

**Figure 1:**
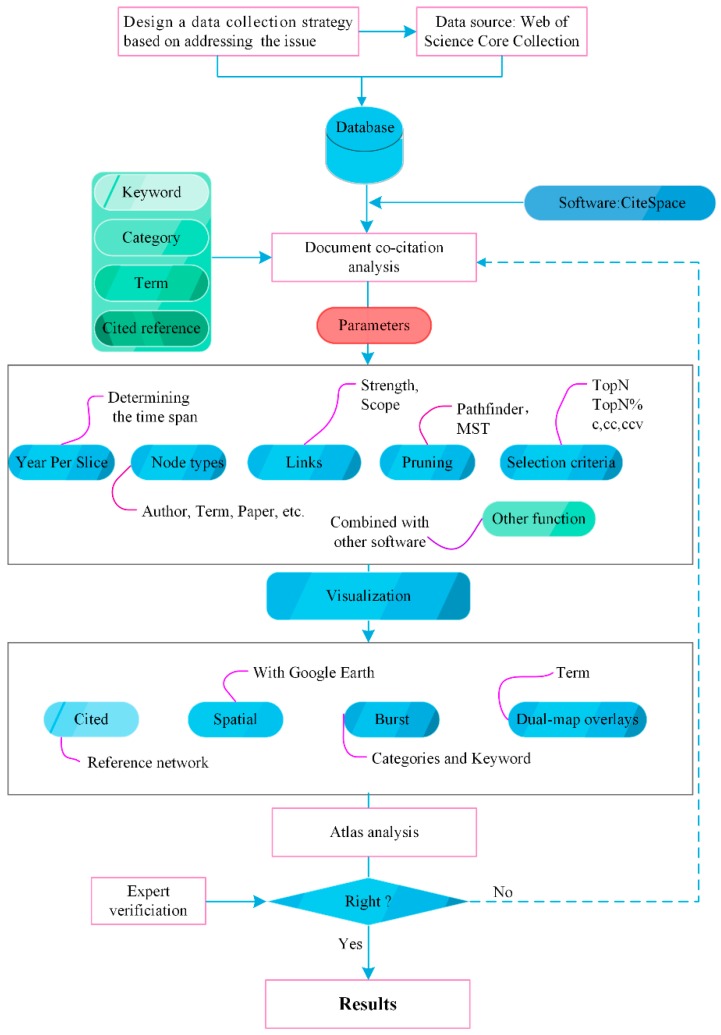
Flow diagram of bibliometric analysis by CiteSpace. Noted that the MST represents the Minimum Spanning Tree.

To detect the emerging trends and new developments in studying disasters after a catastrophic event, this section presents a bibliometric procedure using CiteSpace (Drexel University, Philadelphia, PA, USA) combined with Google Earth (Google, Mountain View, California, USA) (Figure 1). The data collection and the CiteSpace evaluation details are provided in the following subsection.

### 2.1. Data Collection

The bibliographic database of disasters can be created via three steps.

First, the 2008 Wenchuan Earthquake is selected as a time node. At the time of this article, the 2008 Wenchuan Earthquake had occurred almost 10 years ago. The surge of research for this event has basically ended. Therefore, a large number of references could be provided as a basis for the bibliometric analysis.

Second, a data collection scheme is designed. According to Chen et al. [21], there are two types of search datasets: a topic search dataset and a journal search dataset. The former dataset is suitable for analysing interdisciplinary topics. Disasters have significant natural properties (hazard) and social properties (damage). Thus, we select the topic search dataset to investigate new developments in disaster research. Here, the topic includes “Wenchuan Earthquake” and “Disaster”. To compare the differences in disaster research between China and the world, the “Disaster” search includes two types: (1) directly searching for the “Disaster” topic and (2) using “Disaster” as the topic and “China” as the address. Moreover, the time span to detect the change in the study trends is from 1999 to 2017, which is approximately 10 years before (1999–2007) and 10 years after (2008–2017) the earthquake. Last, the data resource is the Web of Science Core Collection because it can provide additional information on references [21].

Finally, the data collection results indicate that there are 3461 records for the topic “Wenchuan Earthquake”. For the topic “Disaster”, there are 10,307 and 55,786 records in China and the world, respectively. The details are shown in Table 1.

### 2.2. CiteSpace

#### 2.2.1. Analysis of Co-Cited References

A co-citation network with a “time slice” overlay will be generated by using CiteSpace, which will then be used to reflect the dynamic changes in disaster research fields [21]. Some landmark references could be extracted from our datasets via this function. These references can describe some popular topics in disaster studies, the evolution of disaster research, the important authors and their academic ideology [22]. Notably, some parameters need to be determined for the abovementioned functions. In this study, the time periods are two years for the topics “Disaster” and “Disaster & China” and one year for the topic “Wenchuan Earthquake”, and the top 100 results are selected. The predefined values in version 5.1. R8SE are used for the other parameters.

#### 2.2.2. Dual-Map Overlay Analysis

A dual-map overlay analysis was developed and embedded into CiteSpace by Chen and Leydesdorff [19]. In the dual-map overlays, the basemap for citations was built based on 10,000 journals [23]. Based on the input dataset, citing trajectories are generated in the dual-map overlay module, which presents the dynamics of previous study activities with cross-disciplines. In others words, the module can describe the citation patterns at the disciplinary level across the entire range of scientific publications for a specific theme [24]. In this study, the dual-map overlay analysis is indispensable for capturing the characteristics of multidisciplinary disaster research.

#### 2.2.3. Burst Detection Analysis

A burst detection algorithm was developed by Kleinberg [25], and it searches for scientific features that have high intensities over finite/limited temporal durations and capture the sharp increases in interest in a specific research field [26]. In addition, this function can serve as a methods of identifying topics or concepts that rose to prominence, were actively discussed for a period of time, and then faded away. In this study, a burst detection algorithm is applied to extract the important categories and keyword for disaster research, and then some of the most active topics are presented to reflect emerging trends in disaster science research.

#### 2.2.4. Geospatial Distribution with Google Earth

The geospatial distribution reflects the outbreak sites and locations of the institutions of authors that have researched a specific theme in a region [20]. This function is not directly available in all versions of CiteSpace. Therefore, Chen [27] provided an approach to visualize the geospatial networks and locations of citations in the R611/05/2007 version of CiteSpace, i.e., the keyhole markup language (KML) files, and revised the KML generator in the 3.8 R23/1/2014 version to increase the precision of the KML results. In addition, the geospatial visualization of publications could be achieved by Google Earth software or an online Google Fusion table. To present the level of focus on disaster research among countries, a geospatial distribution analysis was employed in this study.

## 3. Results

### 3.1. Empirical Characteristics of Publications

**Figure 2:**
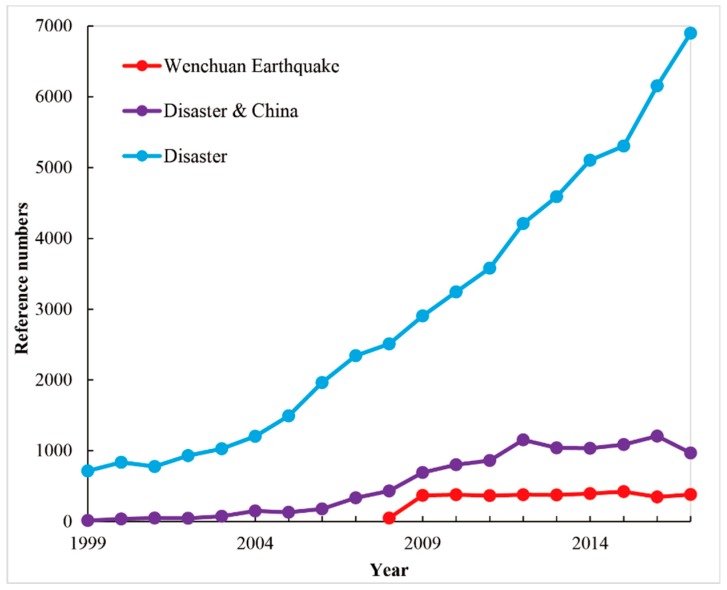
Numbers of disaster research articles from 1999 to 2017.

As shown in Figure 2, the number of articles on the “Wenchuan Earthquake” and “Disaster” has been highlighted. For the topic “Wenchuan Earthquake”, the number of articles significantly increased and surpassed 350 records every year after 2009; however, fewer articles (48 total) were found in 2008 due to the publication time lags because this disaster event occurred on 12 May 2008. Over the past 10 years, studies related to the “Wenchuan Earthquake” were continuous and stable. For the topic of disaster in China, the number of articles was almost consistent with the trend observed for the “Wenchuan Earthquake” topic after 2008, which indicates that the “Wenchuan Earthquake” made a large contribution to the number of disaster research articles. However, there is a remarkable gap in the number of articles between China and the world. The number of articles on disasters in China made a slight contribution to the total number of articles throughout the world. With limited numbers, the influence of these articles will be further detected in the following section, and then the differences in disaster research between China and the world will be compared.

**Figure 3:**
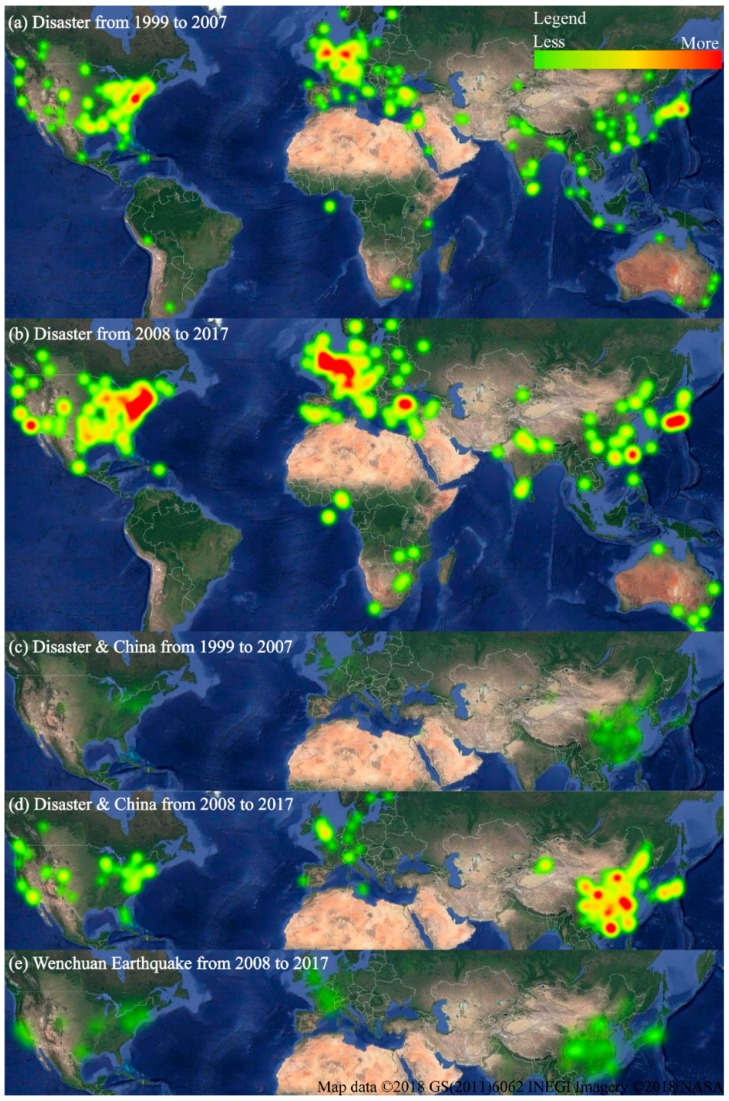
Geographical hotspots of publications related to the “Disaster” topic generated by Google Earth. (**a**) and (**b**) are the spatial distributions of disaster research throughout the world during two periods: 1999–2007 and 2008–2017, respectively. (**c**) and (**d**) are similar to (**a**,**b**) for the “ Disaster & China” topic. (**e**) The spatial distribution of the studies related to the “Wenchuan Earthquake” over the last 10 years.

As shown in Figure 3, both the vertical comparisons of the same topic at different times (two periods: 10 years before and after the 2008 Wenchuan Earthquake) and the horizontal comparisons between topics showed significant differences in the spatial distribution of publications. For the vertical comparisons, although the number of publications related to “Disaster” was significantly higher in 2008–2017 than 1999–2007, the spatial distribution was relatively consistent (Figure 3a,b). Most studies on the “Disaster” topic were published in the Eastern U.S., Western Europe, and East and South Asia. This distinct spatial distribution was already formed in 1999–2007 and was further strengthened in 2008–2017 (Figure 3a,b). For the topic “Disaster & China”, the spatial distribution of the publications was obviously different in the two periods: 1999–2007 and 2008–2017 (Figure 3c,d). From 1999–2007, the few publications were mostly distributed in Eastern and Southern China (Figure 3c). Moreover, few publications have focused on the Eastern U.S. and Southern England (Figure 3c), which means that international cooperation on disaster research was very weak in China before the 2008 Wenchuan Earthquake. From 2008–2017, the number of publications in China was significantly higher than that from 1999–2007 (Figure 3d). Therefore, Southwestern China is a new region of disaster research focus. Moreover, the publications are also extended to Canada, the Midwest U.S., Northern and Southern Europe, and Japan compared with those in 1999–2007, which means that international cooperation remarkably strengthened in China after the 2008 Wenchuan Earthquake.

For the horizontal comparison, the hot spot regions of disaster research throughout the world show that there are obvious regions that closely cooperate with China, both before (Figure 3a,c) and after (Figure 3b,d) the 2008 Wenchuan Earthquake. The publications on research disaster issues in the international regions that cooperate with China accounted for a large proportion of all publications on disasters from 2008–2017 (Figure 3b,d). Finally, although research on the “Wenchuan Earthquake” topic mostly comes from China, hot spot regions in the rest of the world also focus on this topic (Figure 3e).

### 3.2. Emerging Trends and New Developments (1999–2017)

#### 3.2.1. Subject Categories with Citation Burst

**Figure 4:**
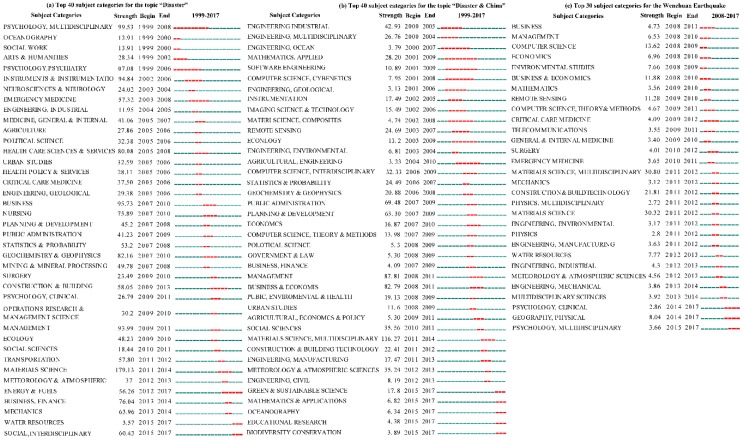
Summary of the subject categories with the strongest bursts (the popularity of one subject increased abruptly over time) for the “Disaster” topic in the Web of Science Core Collection. The blue line is the time interval, the red line segment is the duration of the burst for one subject, the beginning and end represent the boundaries of the time period of the burst, and the strength represents the degree of the burst.

Figure 4 shows that the burst of subjects for the three datasets on the “Disaster” topic obtained from the Web of Science had obvious distinctions. For the “Disaster” topic, we found that the applied studies were focused studying disasters from a global perspective over the past 20 years (Figure 4a). First, the number of publications in these subjects (psychology, humanities, neurosciences, emergency medicine, health policy & services, nursing, and clinical) increased abruptly, indicating that physical and psychological health is an important branch of disaster research. Second, social-economic development and infrastructure were given extensive attention over the past 20 years, e.g., social work, political science, urban studies, business, planning & development, public administration, social sciences, energy & fuels, finance, and transportation. Finally, a few engineering categories could not be ignored, e.g., industrial engineering, geological engineering, and construction & building. In summary, all of these subjects belong to the social aspects of disasters. In other words, the aftermath of disasters was extensively studied throughout the world.

In the topic “Disaster & China”, the fundamental research was given more attention, and the applied studies lagged in China compared with the world (Figure 4b). Before 2008 (i.e., before the 2008 Wenchuan Earthquake), engineering and technology subjects are hot fields of disaster research in China. The engineering subjects included industrial, multidisciplinary, oceanographic, geological, environmental, agricultural engineering. Technology subjects included computer science, interdisciplinary subjects, instrumentation, and remote sensing. These subjects meant that soft adaptive behaviour for responding to disaster risks in China was lacking. After 2008, the applied studies increasingly addressed decreasing the focus on fundamental research. In other words, the social property of disasters has gradually become a hot field of studying disasters, e.g., studying public administration, economic development, laws and policies, and education after disasters. The burst of disaster education reflected the weak awareness of disaster prevention and reduction for Chinese people. Potentially, these improvements could be attributed to the promotion of a special disaster event (e.g., the 2008 Wenchuan Earthquake).

For the “Wenchuan Earthquake” topic, the social property of disasters was an important research field within 10 years after the 2008 Wenchuan Earthquake (Figure 4c). The popular subjects included business, management, economics, medicine, telecommunications, construction, manufacturing, and psychology. The medicine topic included three subcategories: critical care, emergency, and general & internal. The demands for medical care originated from the enormous number of casualties (total: 461,793) in the 2008 Wenchuan Earthquake, which could have potentially driven the advancement of medicine research with related to disasters. Interestingly, a burst of construction & building technology research occurred in the fourth and fifth years after the earthquake, which was driven by the massive demands to rebuild households, infrastructure and industry. The time lag of this burst could have been caused by the article publication cycles. In addition, the inherent characteristics of earthquakes resulted in the subjects related to geography and physics inevitably becoming a hot field of research. In brief, the 2008 Wenchuan Earthquake indeed played an important role in improving the applied disaster research.

The disaster research for applied studies (i.e., adaptive capacity and risk management) in China lagged behind the patterns throughout the world, but a special catastrophic event (the 2008 Wenchuan Earthquake) effectively promoted the advancement of disaster research in China.

#### 3.2.2. Keywords with Citation Burst

**Figure 5:**
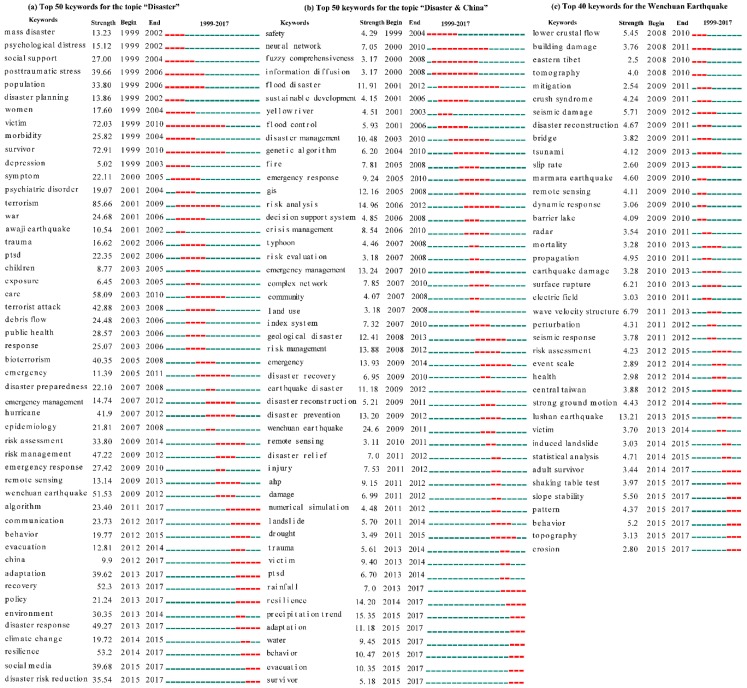
Important keywords with the strongest bursts.

As shown in Figure 5, although dealing with the negative consequences of disasters has always been the most important topic over the past 20 years, the hot topics of disaster research have differed between China and the rest of the world in the same time periods. 

Before 2008, disaster victims and post-disaster medical treatment were an important branch of disaster research for a long time throughout the world (Figure 5a). The keywords “victim” and “survivor” increased abruptly for 11 years from 1999 to 2010. The burst of the keyword “population” lasted for seven years from 1999 to 2006. Certainly, the keywords “women”, “public health”, and “children” were also popular because these sectors were in weak positions when the disasters occurred. These keywords increased abruptly before 2008. For medical treatment, the bursts of important keywords included “psychological distress”, “posttraumatic stress” (the longest duration from 1999 to 2006), “morbidity”, “symptom”, “psychiatric disorder”, “trauma”, “posttraumatic stress disorder (PTSD)”, and “epidemiology”. These kinds of keywords exhibited continuous bursts until 2008 and were rarely cited after 2008.

After 2008, the hot fields of disaster research throughout the world first shifted from disaster victims and medical treatment towards disaster risk management (Figure 5a). This pattern could be reflected by the keywords “disaster preparedness”, “emergency”, “emergency management”, “risk management”, “risk assessment”, “emergency response”, “evacuation”, “adaptation”, “policy”, “disaster response”, “resilience”, and “disaster risk reduction”. These types of keywords were usually used to design macroscopic or microscopic disaster prevention and mitigation strategies. Second, catastrophic events also stirred up a massive wave of disaster research. For example, hurricanes were continuously evaluated with a strength of 41.926 from 2007 to 2012. For destructive earthquakes, the impact of the 1995 Hanshin Awaji earthquake (strength value of 10.54 with burst years from 2001 to 2002) was weaker than the 2008 Wenchuan Earthquake (strength value of 51.53 with burst years from 2009 to 2012) (Figure 5a). Third, research in China frequently appeared from 2012 to 2017, which meant that scholars from China were interested in disaster research (Figure 5a). Finally, the function of using social media to prevent disasters and reducing the damages and the risks of climate change could potentially become a new popular issue in future studies (Figure 5a). From all of the above findings and combined with the results shown in Figure 3a, we believe that the U.S. and European countries had relatively complete systems of medicine research related to disaster before 2008 (i.e., before the 2008 Wenchuan Earthquake) and that the design disaster prevention and mitigation strategies represented new popular fields of disaster research.

In China Before 2008, disaster research focused on disaster risk assessment methods (Figure 5b). For example, the keyword “neural network” was continuously cited from 2000 to 2010. The keywords “fuzzy comprehensiveness” and “information diffusion” were used as a method to assess with the probabilities of frequent risks from 2000 to 2008. The keywords “flood control (2001–2006)”, “genetic algorithm (2004–2010)”, “GIS (2005–2008)”, and “decision support system (2006–2008)” were also related to the disaster risk assessment methods. Certainly, there are other popular keywords. For example, (1) the disaster types included “flood disaster”, “Yellow River”, “fire”, and “typhoon”. The longest time of flood disaster research occurred over approximately 12 years from 2001 to 2012 due to the frequent flooding. (2) Risk management included “disaster management”, “emergency response”, “risk analysis”, “crisis management”, and “risk evaluation”. The field of risk management was popular in China earlier than throughout the world. After 2008, new hot keywords emerged related to the 2008 Wenchuan Earthquake, as shown in Figure 5c, e.g., “emergency”, “disaster recovery”, “earthquake disaster”, “disaster reconstruction”, “Wenchuan Earthquake”, “disaster relief”, “injury”, “damage”, “landslide”, “victim”, “behaviour”, and “survivor”. In other words, a catastrophic event could drive some new hot fields of research. In addition, medical research related to disaster was lagging compared with the patterns in the U.S. and European countries (Figure 5a,b). Only three hot keywords were related to medicine, i.e., “injury”, “trauma”, and “PTSD”. Finally, the hot fields of “resilience”, “adaptation”, and “evacuation” also lagged behind the patterns throughout the world.

For the topic “Wenchuan Earthquake”, hot fields other than the keywords mentioned in the above paragraph were observed (Figure 5c). First, the keywords related to geology included “lower crustal flow”, “tomography”, “slip rate”, “barrier lake”, “surface rupture”, “strong ground motion”, “induced landslide”, “slope stability”, “topography”, and “erosion”. Second, some keywords reflected the negative impacts of earthquakes and became popular, e.g., “bridge”, “propagation”, “electric field”, “mortality”, and “perturbation”. Last, other catastrophic events also exhibited a co-cited relationship with the 2008 Wenchuan Earthquake, e.g., “tsunami”, Marmara earthquake”, and “Lushan earthquake”.

All of the above popular fields of disaster research between China and the world differed in the same time periods, and the 2008 Wenchuan Earthquake drove researchers to increase the disaster prevention and mitigation capabilities in China.

#### 3.2.3. Landmark Articles of Co-Cited References

##### (1) “Disaster” Topic

**Figure 6:**
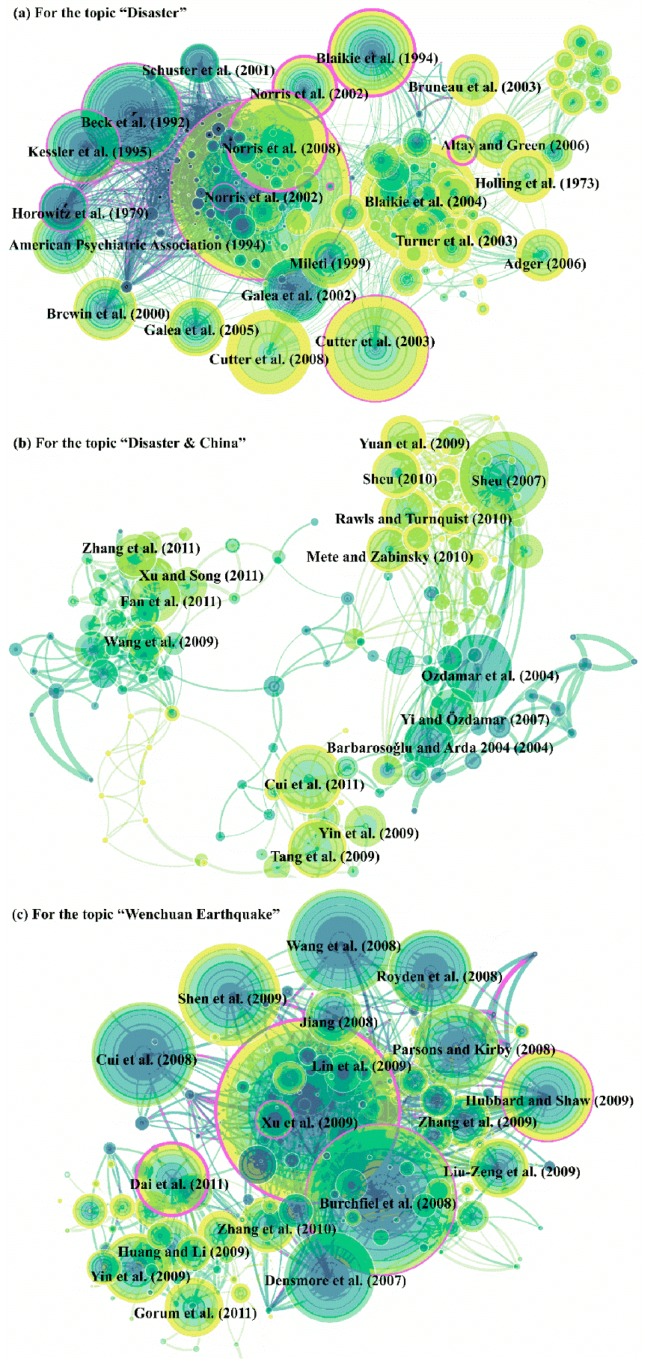
Cited reference network for the “Disaster” topic.

Figure 6 shows the citation patterns with citation tree-rings across multiple time slices, and some important references are extracted and as key citation nodes. For the topic “Disaster”, we extracted 21 important references that were cited more than 200 times from 1999–2017 (Figure 6a). Except for 6 references published before 1999, there were 15 landmark references that could have promoted the advancement of disaster researching after 1999. Only three references were cited less than 1500 times, i.e., Norris et al. [28], Galea et al. [29], and Altay and Green [30]. The remaining references were cited more than 2000 times. According to these references, the emergency medicine related to disaster, vulnerability, resilience, and disaster management fields of disaster science are adequately studied. Mileti [31] designed a method of quantifying how disaster mitigation is viewed, which was used to plan sustainable hazard management projects, and the ultimate aim of this study was to improve the quality of human life, which provided a basic framework for disaster research. Later, scholars further extracted and discussed the vulnerability and resilience of disasters based on the findings in Mileti [31].

For vulnerability, Turner et al. [32] developed a framework to assess the vulnerability of sustainability science by illustrating the emergence of vulnerability research, its approaches and composition, and essential elements for expanding sustainability, which provide a vital theoretical basis for the application of vulnerability analysis to sustainability science. Cutter et al. [33] selected 42 social vulnerability indexes by analysing the danger of environmental hazards and then quantified the spatial patterns of social vulnerability in the U.S. The selection of a vulnerability index was an important contribution by Cutter et al. [33], and this estimation method has been widely employed to assess the vulnerability of other areas. Blaikie et al. [34] described the vulnerability to natural hazards in detail, which included the definition of vulnerability, the principle for selecting a socioeconomic index, an assessment method, and the results. As the most comprehensive monograph for vulnerability, Blaikie et al. [34] was cited more than 7574 times based on the statistics from Google Scholar. Undoubtedly, Blaikie et al. [34] is one of the landmark references for vulnerability studies over the past 20 years. Compared with the antecedent vulnerability to hazards, Adger [35] attempted to capture the vulnerability combined with resilience and adaptation to environmental changes (especially climate changes), which has been widely accepted. With the theory of vulnerability enrichment, vulnerability assessments for each hazard type at different region scales have been carried out.

Bruneau et al. [36] defined the seismic resilience of a community from 4 aspects: materials and engineering science, psychology, sociology, and economics and provided a detailed process for estimating resilience. As a basic tool to assess the post-disaster recovery strength and flexibility of a community, the notion and assessment framework for resilience by Bruneau et al. [36] has been extensively used and has been cited more than 1799 times. Altay and Green [30] provided interfaces with other disciplines via a bibliometric analysis of disaster operation management on the community scale, and this plays a vitally important role in guiding subsequent studies. Cutter et al. [37] developed a new method to assess a resilience framework by adopting different fields and illuminating the conceptual differences and relations among resilience, adaptive capacity, and vulnerability, which further deepens the vulnerability theory. In the meantime, Norris et al. [38] suggested that community resilience, which could serve as a design strategy for disaster readiness projects, must include adaptive capacity, social capital, information and communication, and community competence and then built a theoretical relationship from resilience to adaptation to wellness. This relationship could effectively excavate the extension of resilience. With the theories and approaches mentioned above, case studies on resilience have flourished.

In addition, emergency behaviour from a medical perspective is also a vitally important branch of disaster research. Originally, in an analysis of posttraumatic stress disorder (PTSD), Brewin et al. [39] found that disasters were a vitally important factor. Galea et al. [40] reported on psychological sequelae after the 9.11 terrorist attacks and found that persons that were directly and indirectly affected also suffered from PTSD, and he further contrasted the differences for each PTSD estimation approach [29], which was very useful for selecting suitable methods to assess PTSD in later studies. Moreover, Schuster et al. [41] further found that stress reactions caused by emergency events would result in psychological problems. Importantly, Norris et al. [28,42] illuminated the relationship between disaster characteristics (type, location, observed risk factors, and overall severity of impairment) and outcomes (psychological problems, nonspecific distress, health problems, chronic problems, etc.) in detail according to samples from 60,000 disaster victims. This result provides an important basis for understanding the impacts of disasters on medical research. With these references, the basic framework of emergency medicine related to disaster was built.

##### (2) “Disaster & China” Topic

The number of times that landmark references are cited is significantly lower than the number of times that “Disaster” is cited, and 14 crucial references that were cited more than 100 times were extracted. Six crucial references that originated from non-Chinese scholars [43,44,45,46,47,48] were widely cited by Chinese scholars, which is not considered an advancement for China. The remaining references were related to the 2008 Wenchuan Earthquake, except for Yi and Özdamar [49], who developed a mixed-integer multi-commodity network flow model to guide dynamic logistic programmes during emergency periods after a disaster. Because the mechanisms (dispatching commodities, transferring disaster victims, and logistics coordination, and allocating medical personnel, etc.) are fully considered in this model, and it has been widely applied to address transportation planning problems after disasters.

References related to the 2008 Wenchuan Earthquake mainly considered two issues: geological hazards and mental health. For the geological hazards after the 2008 Wenchuan Earthquake, Yin et al. [50] comprehensively discussed the factors and types of landslide hazards that were triggered by the earthquake and its spatial distribution; Tang et al. [51] fully analysed the growth process and spatial characteristics of the debris flows caused by rainfall; Cui et al. [52] assessed the degree of severity for four types of geo-hazards: rock avalanches, landslides, debris flows, and unstable slopes. For mental health, Wang et al. [53], Xu and Song [54], Fan et al. [55], and Zhang et al. [56] focused on PTSD. The study approaches are similar to those of Norris et al. [28,42] and Galea et al. [4]. These references provide a basis to clearly understand the secondary disaster impacts, and the impacts after an earthquake and are also largely cited. Meanwhile, these references displayed a massive wave of research after the 2008 Wenchuan Earthquake.

##### (3) “Wenchuan Earthquake” Topic

Seventeen crucial references that were cited more than 200 times were extracted, and these references included four categories. The first category was landslide hazards induced by earthquakes. Yin et al. [46] assessed the dynamic changes in landslide hazards over short periods after an earthquake, Dai et al. [13] and Gorum et al. [57] assessed the individual characteristics and spatial distribution of landslide hazards with a large number of samples (more than 56,000) from three years after an earthquake. With changes in the surface environment, landslide scraps have either been newly generated or disappeared in Dai et al. [13] and Gorum et al. [57], which is different from the patterns found in Yin et al. [46].

The second category was geological evolution. Interestingly, the landmark references for this issue were mainly focused on by non-Chinese scholars. Burchfiel et al. [58] amply discussed the geological and geophysical contexts of the earthquake. Royden et al. [59] believed that the 2008 Wenchuan Earthquake resulted from the rapid eastward flow of the deep crust in the Tibetan Plateau, as indicated by a tectonic reconstruction analysis. Parsons et al. [60] found that the rearrangement of stresses in the crust resulted in subsequent damaging earthquakes. Wang et al. [61] revealed the hypocentre process by combining the inverted teleseismic waveforms and local coseismic displacement. Hubbard and Shaw [62] found that the crust uplift in the Longmen Shan and Tibetan Plateau resulted from the earthquake by geologic surveys.

The third was the surface rupture of the earthquake. Zhang et al. [63] analysed the spatiotemporal rupture process of the earthquake in detail. Shen et al. [64] revealed a slip process in the rupturing belt. Liu-Zeng et al. [65] and Lin et al. [66] detected the east-west crustal shortening on oblique, parallel thrusts along the eastern edge of the Tibet Plateau. Based on the basic information mentioned above, Zhang et al. [67] and Xu et al. [68] discussed the geologic changes after the earthquake, including oblique, high-angle, listric-reverse and faulting, and strain changes.

Last, the development and distribution of geologic hazards that were triggered by the earthquake were also closely evaluated [69,70]. With these references, we could clearly understand the geologic changes and the relative secondary hazards after the earthquake.

The 2008 Wenchuan Earthquake indeed triggered a series of studies that presented finding consistent with the results of Liu and colleagues [71], and scholars paid more attention to analysing geologic changes and assessing secondary hazards after the earthquake. Meanwhile, the number of references that originated from the U.S. and European countries was significantly higher than the number that originated from China, i.e., the quality of references published by the U.S. and European countries was high. Interestingly, although risk change and recovery processes for disasters were focused on before the 2008 Wenchuan Earthquake, references that studied damage and loss assessments were not largely cited. In other words, this issue has not received much attention for this event. Notably, the theory and application of building disaster prevention and mitigation capacities are greater in the U.S. and European countries than China. The theory and methods for estimating vulnerability and resilience and designing emergency plans in China mainly came from the U.S. and European countries. Therefore, disaster research in China lagged behind that in the U.S. and European countries, and large investments are needed to improve or enhance the theoretical basis and practical application of disaster science in China.

#### 3.2.4. Interdisciplinary Level with Dual-Map Overlays

##### (1) “Disaster” Topic

**Figure 7:**
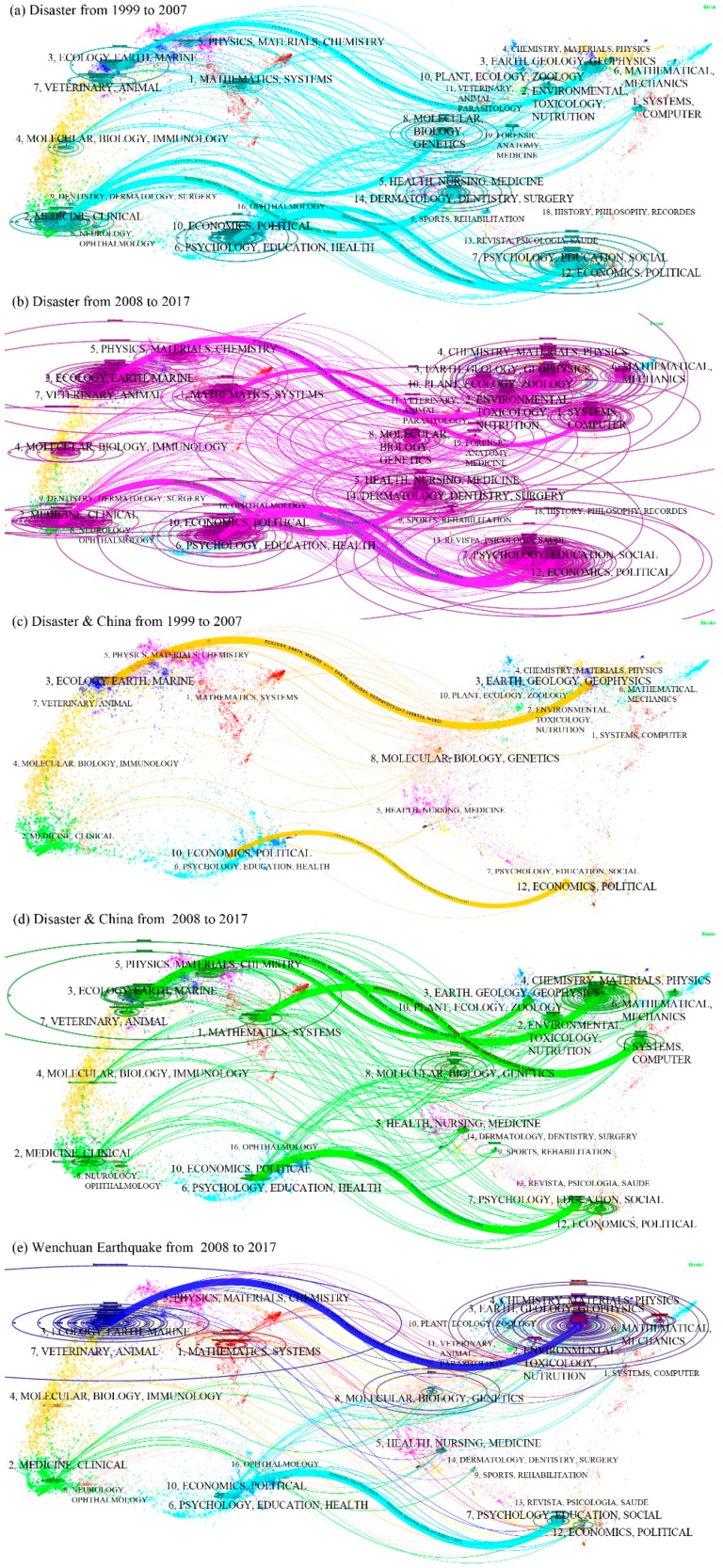
Study activities for the “Disaster” topic with cross-disciplines over the past 20 years. The left side and right side are the citing journals and the cited journals, respectively; each dot is a journal, and the line provides a function of the link that connects the citing journal and the cited journal. The longitudinal axis of the ellipse represents the numbers of articles published in one journal, and the horizontal axis of the ellipse represents the corresponding author number. Each label represents a discipline. Refer to Chen and Leydesdorff [19] for the detailed theories and techniques. With these data, citing behaviours can be clearly described.

As shown in Figure 7, a new interdisciplinary discipline emerged from 2008–2017, and it is different from the disciplines observed from 1999–2007. Before 2008, 6 groups of citations were observed for the majority of disciplines throughout the world (Figure 7a). In detail, two groups of internal citation patterns were observed, i.e., (i) the economics & political discipline and (ii) the psychology, education & social discipline, and four groups of transdisciplinary citation patterns were observed, i.e., (i) the Earth, geology & geophysics discipline combined with the ecology, Earth & marine discipline, (ii) the molecular, biology & genetics discipline combined with the medicine, medical & clinical discipline, (iii) the health, nursing & medicine discipline combined with the medicine, medical & clinical discipline, and (iv) the health, nursing & medicine discipline combined with the psychology, education & social discipline. The last 4 groups could reflect a paradigm shift for disaster science. As with (ii) and (iv), we believe that the medicine, medical & clinical discipline was reinforced by absorbing the theories and applications of the molecular, biology & genetics discipline and the health, nursing & medicine discipline. With (iii) and (iv), we could tell that the health, nursing & medicine discipline provided a basis for the development of the medicine, medical & clinical discipline and the psychology, education & social discipline.

After 2008, a new trend of journal citations emerged (Figure 7b). First, the transdisciplinary research of disaster science was further reinforced. For example, the number of investigators focusing on the Earth, geology & geophysics combined with the ecology and Earth & marine disciplines increased rapidly (see the horizontal axis of the ellipse in Figure 7b), and the number of publications rapidly increased (see the longitudinal axis of the ellipse in Figure 7b) compared with the patterns in Figure 7a. The psychology, education & health discipline substantially absorbed the ideology of the economic & political discipline. Second, a new relationship of transdisciplinary citations was built. For example, the systems & computer discipline was largely cited by the mathematics & systems discipline. Third, there was a weakening trend for the medicine, medical & clinical discipline compared with the trend from 1999–2007. The citation relationship was limited within this discipline. Fourth, some popular interdisciplinary research topics that were evaluated from 1999–2007 continued from 2008–2017 and could be continued in the future. For example, the research that focused on the economic & political and psychology, and education & social disciplines increased, and the number of related publications also increased. Thus, an obvious new trend for the study of disaster science obviously emerged throughout the world.

##### (2) “Disaster & China” Topic

In China, the transdisciplinary citation pattern was obviously weaker than compared with that throughout the world, although it increased over the past 20 years. Before 2008, there were only two groups of mass citations (Figure 7c). One group was from the Earth, geology & geophysics combined with the ecology, Earth & marine discipline. Another group was the psychology, education & social discipline. Notably, these citation patterns were consistent throughout the world. While there were less than four groups throughout the world (Figure 7a,c); i.e., the medicine & clinical discipline cited (1) the molecular, biology & genetics discipline and (2) the health, nursing & medicine discipline; (3) the health, nursing & medicine discipline was also cited by the psychology, education & health discipline, and (4) the internal citations of the economics & political discipline.

After 2008, some new transdisciplinary citation patterns emerged (Figure 7d). First, two outstanding groups of transdisciplinary citations were formed, i.e., the systems & computer discipline, which was cited by the mathematics & systems discipline, and the plant, & zoology discipline, which was cited by the ecology, Earth & marine discipline. Second, several transdisciplinary citations were formed with weakly linked citations. For example, the articles of the Earth, geology & geophysics discipline were cited by the economics & political discipline, which meant that the economics research also involved some geography issues. The articles of the medicine & clinical discipline cited articles from the psychology, education & social discipline. In summary, the interdisciplinary level of disaster research was significantly increased after 2008 in China.

##### (3) “Wenchuan Earthquake” Topic

Two outstanding groups and several uncommon groups of transdisciplinary citations have been detected in the “Disaster & China” topic (Figure 7e). First, the interdisciplinary research from the Earth, geology & geophysics discipline and the ecology, Earth & marine discipline made a significant contribution to disaster research in China because articles related to this event were published in this interdisciplinary field. Fewer studies have been performed in the psychology, education & social discipline. Second, the articles of the ecology, Earth & marine discipline also cited literature from the psychology, education & social and the economics & political disciplines. Third, the medicine & clinical discipline was related to the Earth, geology & geophysics discipline, the systems & computer discipline, the psychology, education & social discipline, and the economics & political discipline. Other detailed transdisciplinary citation patterns were not introduced.

Thus, the transdisciplinary level is increasingly obvious throughout the world and in China over time. This interdisciplinary research is in accordance with the characteristics of disaster science as a synthetic discipline and will be strengthened in the future.

## 4. Conclusions

To detect the massive waves of disaster research created by representative catastrophic events, this study takes the 2008 Wenchuan Earthquake as a time node and uses scientific bibliometric visualization to identify hot points of changes to disaster study fields before and after the 2008 Wenchuan Earthquake. Some interesting trends and new developments are summarized as follows.

First, with the increase in interest in disaster science investigations, the number of publications from the U.S. and European countries and China increased significantly over the past 20 years. As a special catastrophic event, the 2008 Wenchuan Earthquake was widely evaluated throughout the world and resulted in a large number of studies. Moreover, the event also remarkably strengthened international cooperation to build a disaster science discipline.

Second, the social properties of disasters, including medical treatment of disaster victims, disaster impacts on social-economic development, and engineering categories, were more commonly evaluated by the U.S. and European countries after the event compared with that over the past 20 years and gradually became hot research topics after the 2008 Wenchuan Earthquake in China. Certainly, the adaptive capacity of recovery and risk management of disaster response is also a new popular issue for disaster research.

Third, disaster research shifted from fundamental research to applied studies for China after the 2008 Wenchuan Earthquake. Although these applications (mental health of disaster victims and reconstruction) were also addressed they were relatively weak, and fundamental studies of hazards (geological disaster and surface change) remain the most concerning issues after an earthquake.

Fourth, the references from China are cited frequently less than those from the U.S. and European countries, and the level of innovation for disaster research in China is significantly weaker than that in the U.S. and European countries. Although the transdisciplinary level of disaster research has been heightened worldwide over the past 20 years, the level in China is still weaker than that in the U.S. and European countries. Thus, reinforcing the theoretical construction of disaster science in China is indispensable.

Last, this study is preliminary work and has provided valuable information related to disaster study fields by bibliometric methods. However, some limitations were still observed. (i) Although references from the Web of Science Core Collection are of own better quality and can be effectively visualized by CiteSpace software, this study is not reflective of studies published in other languages, such as Chinese, French, Japanese, German, and Spanish. (ii) Although we found some new disaster-related hotspot subjects, the implications of those subjects need further analysis. Thus, deeper insights from domain experts are necessary to better understand disaster science in the future.

## Figures and Tables

**Table 1:** Summary of the topics searched for in our database.

Dataset	Duration	Records
Wenchuan Earthquake	2008–2017	3461
Disaster & China	1999–2007	1023
	2008–2017	9285
Disaster	1999–2007	11,292
	2008–2017	44,516
